# Outcomes of vitrectomy for chorioretinitis sclopetaria following blast-related ocular trauma

**DOI:** 10.1186/s40942-025-00674-5

**Published:** 2025-04-22

**Authors:** Nadiia Ulianova, Oksana Sidak-Petretska, Nataliia Bondar

**Affiliations:** State Institution, “The Filatov Institute of Eye Diseases and Tissue Therapy of NAMS of Ukraine”, Frantsuzkiy Bulvar, 49/51, Odesa, 65015 Ukraine

**Keywords:** Chorioretinitis sclopetaria, Vitrectomy, Blast ocular trauma, Retina

## Abstract

**Background:**

To analyse the anatomical and functional results of pars plana vitrectomy in patients with Chorioretinitis Sclopetaria caused by severe combat-related ocular trauma.

**Methods:**

This retrospective, observational study involved 24 cases of pars plana vitrectomy in patients with Chorioretinitis Scleropetaria following combat-related ocular trauma. Best-corrected visual acuity and retinal reattachment were studied. The data were analysed via quantitative and categorical correlation analyses, as well as logistic regression models.

**Results:**

Postoperative best-corrected visual acuity improved in 18 patients (75%) but remained unchanged in 5 patients (20.8%). In 1 patient (4.2%), best-corrected visual acuity deteriorated due to the development of traumatic optic neuropathy. Retinal detachment was noted in 13 patients, whereas a macular hole was present in 5 patients. In 2 patients, both retinal detachment and macular holes were diagnosed simultaneously. After pars plana vitrectomy, retinal reattachment was achieved in 23 patients (95.8%). In one case, reattachment was unsuccessful. The localization of Chorioretinitis Sclopetaria was significantly associated with the final best-corrected visual acuity, with the best surgical outcome observed in patients with Chorioretinitis Sclopetaria located in the inferior sector of the fundus (*p* < 0.05). The outcome of pars plana vitrectomy for Chorioretinitis Sclopetaria with concomitant retinal detachment is significantly better when the procedure is performed earlier following blast injury.

**Conclusions:**

Chorioretinitis Sclopetaria following blast ocular trauma is characterized by a significant, persistent, best-corrected visual acuity decreasing, a high frequency of vitreous haemorrhages, macular holes, and retinal detachment. Pars plana vitrectomy in Chorioretinitis Sclopetaria has shown considerable effectiveness in improving visual function, retinal reattachment, and macular hole closure in patients with blast-related ocular trauma.

**Clinical trial number:**

Not applicable.

## Introduction

Chorioretinitis Sclopetaria (CS) is a rupture of the choroid and overlying neurosensory retina caused by a high-velocity projectile that is adjacent to but does not penetrate the globe [1]. CS is a relatively rare condition that occurs when the orbit is damaged by a bullet, shotgun pellet, paintball projectile, ball, or a high-velocity foreign body that has bounced off the surface of a hard object when it is struck with a hammer [2–4]. Even less common are cases of CS developing due to trauma as a result of exposure to fireworks or hydraulic shock [5, 6].

Current ballistic concepts of the mechanisms of the damaging effect of gunshot wounds indicate that rupture, compression, and destruction of tissue by a bullet lead to the formation of a permanent wound cavity identical to the diameter of the bullet [7]. In turn, a temporary wound cavity is formed by shock waves propagating perpendicular to the path of the bullet [8]. In this case, inelastic and loose tissues of the body are damaged to a greater extent than elastic tissues [9]. If we consider the mechanism of CS occurrence in gunshot trauma of the orbit without damaging the integrity of the globe, it becomes obvious that the sclera, which is a more elastic layer, is less damaged than the underlying choroid, Bruch’s membrane, and retina [10]. Tissue damage in blast injuries is particularly severe because of the simultaneous impact of several groups of factors on the body’s tissues. When high-velocity foreign bodies form as a result of an explosion entering the orbit, the pathophysiological mechanisms for the development of CS arise not only as a result of the local damaging effect of the projectile but also as a result of the impact of the shock wave, both locally on the periorbital region and on the human body as a whole.

Owing to the low incidence of this disease, there is no consensus on the management of patients with CS [4]. There are two opposing approaches. One of them involves conservative monitoring; the second involves pars plana vitrectomy (PPV) [1]. In this case, the functional prognosis, according to most authors, depends on the location of the CS, as well as the severity of maculopathy and fibrovascular proliferation [3]. Importantly, these conclusions are related mainly to gunshot injuries. In cases of CS caused by blast trauma, the globe is subjected to a comparatively greater number of damaging factors, which may result in more severe clinical manifestations. This necessitates a refinement of the surgical treatment strategy specifically for CS resulting from blast injuries. However, systematized data on the surgical management of CS caused by blast trauma are currently lacking.

The intensification of military conflicts in Ukraine has led to an increase in the number of combat-related injuries to the orbit and globe, including the development of CS [11, 12]. According to our observations, CS caused by blast-induced ocular trauma is characterized by a marked and persistent reduction in visual function, a high incidence of massive vitreous hemorrhages, macular tears, and the development of retinal detachment [12]. Therefore, a detailed analysis of each case of CS in the setting of modern severe blast trauma is crucial for a deeper understanding of the specific clinical manifestations and for making an informed choice of the optimal management strategy for this category of patients.

*The purpose* of our study was to analyse the anatomical and functional results of PPV in patients with CS caused by severe combat-related ocular trauma.

## Methods

### Patients

A retrospective study included an analysis of 24 cases (24 eyes) of CS secondary to closed-globe injury following an explosion. Among the patients, 23 were male, and 1 was female. The age of the patients ranged from 24 to 59 years. The main criterion that distinguished CS from other types of traumatic chorioretinopathy was a closed-globe injury accompanied by a penetrating orbital fragment wound. Surgical treatment of all patients was performed at the Department of Post-Traumatic Eye Pathology of the State Institution “The Filatov Institute of Eye Diseases and Tissue Therapy of NAMS of Ukraine” from May 2022–October 2024. Among them, 22 patients sustained blast injuries, while 2 suffered gunshot wounds to the orbit. A total of 21 patients were diagnosed with nonperforating shrapnel injuries of the orbit, whereas in three patients, the orbital wound was perforating. In 14 patients (58.3%), the presence of a foreign body in the orbit was noted at the time of hospitalization; in 7 patients (29.2%), the foreign body had been removed during previous stages of medical care. The entry site was predominantly located in the unilateral periorbital region. The wound trajectory passed through the maxilla in 3 patients, through the frontal bone in 2 patients, through the temporal bone in 2 patients, and through the eyelids and soft tissues of the periorbital region in the remaining patients. In 1 patient, orbital injury resulted in a transpalpebral entry wound on the contralateral side. In 1 patient, there was an open craniocerebral injury with a fracture of both frontal bones and a foreign body in the anterior cranial fossa.

All patients had concomitant traumatic brain injuries and had previously undergone treatment in neurosurgical and neurological departments. After stabilization of vital functions, patients were admitted to the ophthalmology department of the institute for further management.

All patients underwent a comprehensive ophthalmological evaluation, including visual acuity assessment, biomicroscopy, ophthalmoscopy, and ultrasonography, before being indicated for surgical intervention.

### Surgical treatment

In all the presented cases, a 25-G three-port PPV was performed, including combined phacovitrectomy in 10 patients (41.7%) with significant lens opacity. The main indications for vitrectomy were the presence of vitreous haemorrhage, retinal detachment, and/or macular holes. The method of tamponade of the vitreous cavity was selected on the basis of the presence or absence of retinal detachment and the number and location of retinal tears. In 50% of the cases, tamponade was performed with silicone oil, whereas in the other 50%, an air‒gas mixture was used. During PPV in patients with CS, posterior hyaloid membrane removal was performed. A noteworthy feature was the strong adhesion of the posterior hyaloid membrane in the sclopetarian zone. Internal limiting membrane (ILM) peeling was performed in all patients with a macular hole. In two patients with large-diameter macular holes (greater than 500 μm), the inverted ILM flap technique was applied. Primary ILM peeling is also routinely performed in all cases where the sclopetarian lesion is located temporally, which, in our opinion, helps to reduce tangential retinal traction in the macular area and may lower the risk of epiretinal fibrosis development in the late posttraumatic period.

**Fig. 1:**
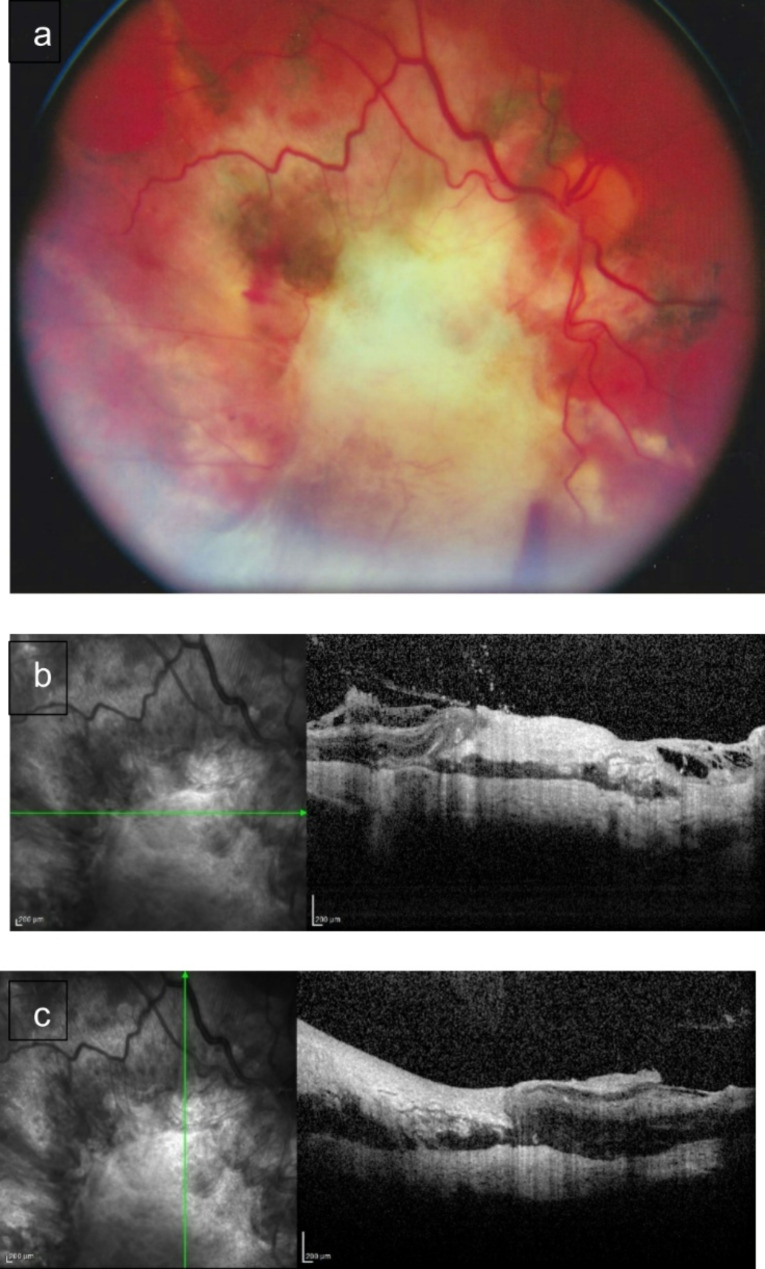
Fundus photo of the central CS (**a**). Optical coherent tomography of the macula of a patient with CS (**b**, **c**)

The criteria for assessing the effectiveness of surgical treatment were the dynamics of changes in BCVA, retinal reattachment, and the morphology of the chorioretinal complex according to optical coherence tomography (Fig. 1). The total follow-up period for patients was 12 weeks.

The following factors were analysed for their impact on surgical outcomes: the presence of retinal detachment, the presence of retinal tears, the time interval between trauma and surgical intervention, the presence of orbital wall fractures, the location of the sclopetarian lesion, and the presence of an intraorbital foreign body. On the basis of the vitrectomy results, patients were divided into two groups: (1) those with improvement (visual acuity improved after vitrectomy) and (2) those without improvement or with worsening (visual acuity either remained unchanged or decreased following vitrectomy).

### Statistical analysis

Descriptive statistics and data visualization methods were used to identify key characteristics of the sample and assess the distribution of variables. The biserial correlation coefficient was used to study the degree of association of pairwise statistical relationships between numerical and binary variables. To assess the degree of pairwise relationships between binary variables, two-way contingency tables and related coefficients were used: association coefficients, Cramér’s V, Fisher’s exact test, Barnard’s exact test, and Boschloo’s exact test. Logistic regression models were also used to assess the degree of influence of variables on the surgical outcome, and the significance of the model coefficients was assessed. The significance of the results was estimated at alpha = 0.05.

## Results

The BCVA in patients upon admission for treatment ranged from no light perception (NLP) to 20/200 (0.1): NLP in 1 patient (4.2%), light perception (LP) with inaccurate light projection in 11 patients (45.8%), LP with accurate light projection in 5 patients (20.8%), vision worse than 20/400 (0.05) in 6 patients (25%), and vision from 20/400 (0.05) to 20/200 (0.1) in 1 patient (4.2%). The final BCVA varied from NLP to 20/40 (0.5): NLP persisted despite treatment in 1 patient (4.2%), LP with inaccurate light projection remained in 5 patients (20.8%), vision ranged from 20/2000 (0.01) to 20/200 (0.1) in 12 (50%), and greater than 20/200 (0.1) in 6 (25%).

After vitrectomy, visual acuity improved in 18 patients (75%), remained unchanged in 5 (20.8%), and in one of these patients, despite a satisfactory anatomical result, it was impossible to achieve visual improvement due to optic nerve avulsion. In 1 patient (4.2%), visual acuity was decreased due to the traumatic optic neuropathy.

Among the intraoperative complications, we observed intraoperative bleeding in one patient, which occurred in a patient with retinal vessel avulsion. In another case, a patient with a firmly adherent posterior hyaloid membrane at the site corresponding to a large choroidal rupture developed an iatrogenic retinal tear along the edge of the CS lesion.

Different locations of the CS were established in patients. CS was detected in the inferior sector of the fundus in 11 patients (45.8%), in the nasal sector in 3 patients (12.5%), temporal in 3 patients (12.5%), superior in 1 patient (4.2%), parapapillary in 1 patient (4.6%), in the macula in 4 patients (16.7%), and involving more than 2 sectors in 1 patient (4.6%). The location of the CS was significantly associated with the final BCVA, with the best surgical outcome observed in patients whose CS was located in the inferior sector of the fundus (*p* < 0.05) (Table 1).

**Table 1:** Coefficients of the logistic regression model for surgical success depending on the location of the CS

Location CS	coef	Std err	z ^a^	*p*>|z|	[0.025 *	0.975] ^*^
lateral	-0.6931	1.225	-0.566	0.571	-3.094	1.707
macula	-1.0986	1.155	-0.951	0.341	-3.362	1.165
nasal	25.4266	1.92e + 05	0.000	1.000	-3.76e + 05	3.76e + 05
inferior	2.3979	1.044	2.296	0.022	0.351	4.445
parapapillary	24.0157	1.64e + 05	0.000	1.000	-3.21e + 05	3.22e + 05

^a^– z-score of the model coefficient; ^*^ - confidence interval for the model coefficient

Retinal detachment was noted in 13 patients, whereas a macular hole was present in 5 patients. In 2 patients, both retinal detachment and macular hole were diagnosed simultaneously. After PPV, retinal reattachment was achieved in 23 patients (95.8%). In one case, reattachment was unsuccessful.

The coefficients of the relationship between the presence of retinal detachment and the success of the surgery were as follows: the association coefficient was 0.517, Cramér’s V was 0.145, Fisher’s exact test statistic was 3.143 (*p* = 0.357), Barnard’s exact test statistic was 1.183 (*p* = 0.296), and Boschloo’s exact test statistic was 0.239 (*p* = 0.296). There is a weak association between the surgical outcome and the presence of retinal detachment; within this sample, the relationship is statistically insignificant, but its confirmation can also be expected as new data are accumulated.

When the influence of the presence of a macular hole on the success of surgery was assessed, the association coefficient was 1.0, Cramér’s V was 0.178, Barnard’s exact test statistic was 1.451 (*p* = 0.17), and Boschloo’s exact test statistic was 0.202 (*p* = 0.2). There is a weak association between surgical outcome and the presence of a macular hole; within this sample, the relationship is statistically insignificant, but its confirmation can be expected as new data accumulate and the sample size increases.

Surgical treatment was performed at different time intervals after blast injury, ranging from 10 to 286 days. To study the influence of the time elapsed after injury on the success of the surgery, a quantitative correlation analysis was used with the biserial correlation coefficient. The obtained value was *r*=-0.287, *p* = 0.174. This indicates a weak inverse relationship. Consequently, there was a tendency toward better functional results in patients who underwent PPV earlier after injury. However, the result was statistically insignificant for the initial sample size at the standard significance level of 5%.

No statistically significant relationships were found between patient age, the presence of an orbital foreign body, or the presence of orbital fractures and vitrectomy outcomes.

In the next stage of the study, a multivariate analysis was performed to assess the potential combined influence of several factors on the surgical outcomes of CS: the time interval between trauma and surgery, the location of the sclopetarian lesion, the presence of an intraorbital foreign body, the presence of orbital wall fractures, and the presence of retinal detachment or retinal tears (Table 2).

**Table 2:** Coefficients of the multivariate logistic regression model for surgical success depending

Variable	coef	Std err	z ^a^	*p*>|z|	[0.025 *	0.975] ^*^
time interval after blast injury	-0.7111	0.817	-0.871	0.384	-2.312	0.889
foreign body in the orbit	-0.1126	0.854	-0.132	0.895	-1.787	1.562
orbital wall fracture	0.4591	0.700	0.656	0.512	-0.913	1.832
retinal detachment	0.9287	0.672	1.381	0.167	-0.389	2.247
macular hole	1.4237	1.005	1.416	0.157	-0.547	3.394
localization of CS	1.2892	0.717	1.798	0.072	-0.116	2.694

^a^– z-score of the model coefficient; ^*^ - the confidence interval for the model coefficient

As a result of the analysis, negative logistic regression coefficients were obtained for the time interval between trauma and surgery, as well as for the presence of an intraorbital foreign body. This finding indicates that both longer intervals before surgery and the presence of an intraorbital foreign body potentially reduce the effectiveness of vitrectomy. On the other hand, timely vitrectomy in patients with retinal detachment and retinal tears (positive logistic regression coefficients) may achieve the best possible BCVA outcomes. However, when the combined influence of these factors was considered, the results did not reach statistical significance. Notably, multivariate analysis confirmed a statistically significant association between the functional outcome of surgery and the location of the sclopetarian lesion (*p* = 0.072).

In addition, the potential combined influence of several factors on BCVA improvement after surgery was considered, namely, the following factor combinations were analysed: the time interval after injury and retinal detachment, the time interval after injury and macular hole, the localization of the CS and the presence of retinal detachment, and the localization of the CS and macular hole. For these combinations of factors, the corresponding logistic regression models were built, and a statistical assessment of the significance of their coefficients was carried out.

As mentioned above, when the influence of retinal detachment on surgical outcomes was assessed, no statistically significant changes were found, although a trend was observed. However, the analysis revealed a statistically significant logistic regression coefficient of 1.794 (*p* < 0.05) for the combination of two factors: the time from trauma to vitrectomy and the presence of retinal detachment. In other words, the outcomes of PPV for CS with associated retinal detachment are significantly better when the procedure is performed earlier after blast injury (Table 3).

**Table 3:** Coefficients of the logistic regression model for surgical success depending on the time after injury and the presence of retinal detachment

Variable	coef	Std err	z	*p*>|z|	[0.025	0.975]
time after injury	-0.0018	0.006	-0.308	0.758	-0.013	0.010
presence of retinal detachment	1.7943	0.824	2.177	0.029	0.179	3.410

In turn, no significant relationship was found between the results of vitrectomy and the combination of factors: time after injury and the presence of a macular hole. The construction of logistic regression models for the combination of the location of the CS and the presence of retinal detachment or a macular hole revealed that in both the first (logistic regression coefficient 1.5153; *p* < 0.05) and the second (logistic regression coefficient 2.3026; *p* < 0.05) cases, the best functional outcomes of surgical treatment were observed when the CS was located in the inferior part of the fundus.

## Discussion

Our study revealed a relatively greater incidence of retinal detachment in the CS caused by blast-related ocular trauma than previously described in the CS of other etiologies. Thus, a retrospective analysis by Takkar B. et al. of 6 cases of CS revealed the development of retinal detachment in only 1 out of 6 cases, including 1 patient out of 3 cases of blast injury. In turn, among gunshot wounds to the orbit, retinal detachment was not observed in patients in this group [13]. The study by Ludwig C.A. et al., who examined 6 new cases of CS in patients at Stanford University along with a series of previously published cases, also revealed that the incidence of retinal detachment was 11.5% [1]. In our study, the incidence of retinal detachment was 54.2%, which, in our opinion, can be attributed to more significant eye damage in blast injuries, namely, the impact of not only a high-velocity projectile in the orbit but also the shock wave effect on the membranes of the globe, as well as possible concussive forces resulting from body displacement and impact with hard surfaces. Severe vitreous haemorrhages and subretinal and subchoroidal haemorrhages are quite common and occur in more than half of patients (62.3%), as evidenced by multiple publications by different researchers [1]. Among the patients in our study with blast-related injuries, the incidence of vitreous hemorrhages was 83.3%, which could also be attributed to more severe retinal vascular damage, avulsions, and ruptures resulting from blast-related traumatic forces. The incidence of macular holes, according to published data, is 11.5% [1]. In our study, the incidence of macular holes was 20.8%. Thus, it should be noted that in CS resulting from orbital shrapnel injuries due to blast trauma, conditions leading to low initial visual acuity occur more frequently than after gunshot wounds and serve as a key factor in the decision-making process regarding PPV.

The PPV we performed in all patients demonstrated favourable outcomes, including retinal reattachment and stabilization, as well as successful macular hole closure. The presence of pronounced vitreous opacities did not allow for preoperative detection of the macular hole. In all cases, the diagnosis was confirmed intraoperatively after the removal of pathologically altered, opaque vitreous and haemorrhages. The high incidence of macular holes in the CS associated with blast injury, coupled with the inability to detect them preoperatively, should heighten a surgeon’s suspicion when planning surgery for this patient group. Earlier concepts of CS pathogenesis suggested that the risk of retinal detachment in such injuries was low due to retinal-choroidal adhesions. However, subsequent studies have reported entire case series of retinal detachment in the CS, leading to some modifications in surgical approaches [14]. From our point of view, CS resulting from blast injury is characterized by greater severity and more extensive retinal pathology, which supports the indication for PPV. Given the high degree of proliferative changes in the CS, we believe that PPV with removal of the posterior hyaloid membrane and primary internal limiting membrane peeling in the macula is justified, regardless of the presence of a macular hole. This approach is especially warranted in cases where the CS is located temporally, as subretinal fibrosis formation in the choroidal rupture zone, along with chorioretinal scarring, exposes the macula to significant tangential surface traction. Based on the biomechanical properties of the retina, prolonged traction can lead to the accumulation of critical damage, contributing to layer disorganization, even in cases where a macular hole is not initially present following trauma [15].

In contrast to gunshot injuries, blast-related ocular trauma involves multiple damaging factors that act simultaneously on the globe, including mechanical impact from fragments, the blast wave itself, bodily concussion, and thermal effects. In our view, not only the intensity of each of these components but also the direction of force application relative to the sagittal axis of the eye plays a significant role in blast-related injuries. Multidirectional and high-intensity forces generated by fragments and the blast wave may lead to more extensive hemorrhages and subsequently larger areas of fibrosis. This may also account for the higher incidence of retinal detachment and macular tears observed following blast-induced ocular trauma.

The patterns we identified indicate that there is no relationship between the results of surgical treatment and the age of patients, the time after injury, the presence of an orbital fracture, or the presence of a foreign body in the orbit, which is generally consistent with published data [1] and indicates that there are no grounds for removing orbital shrapnel. In turn, the analysis of the surgical results revealed that in cases of retinal detachment and the presence of a macular hole, PPV has a positive effect on the anatomical and functional outcomes. A statistically significant increase in BCVA after PPV compared with baseline was noted in patients with retinal detachment. However, when the influence of the time interval between injury and surgery is assessed, it can be concluded that an increase in time negatively impacts the functional outcome of PPV in the presence of retinal detachment and does not affect it in the case of a macular hole.

Our study revealed that the location of the CS affects the functional outcome of PPV. Given the anatomy of the orbit, foreign bodies can most often enter from the temporal side and inferiorly. The most favorable functional outcome, which consisted of maintaining or improving visual acuity, was observed with the inferior localization of the choroidal rupture in both patients with retinal detachment and patients with a macular hole. This may be due to the fact that subretinal haemorrhages located in the inferior parts of the fundus are distant from the optic nerve and macula and are not subject to hydrostatic displacement towards the fovea, compared to the superior localization. In turn, macular and temporal CS are accompanied by the development of severe macular fibrosis and are associated with worse functional outcomes.

## Conclusion

Chorioretinitis Sclopetaria following blast ocular trauma is characterized by a significant, persistent decrease in visual acuity; a high frequency of massive vitreous haemorrhages and macular holes; and the development of retinal detachment. Pars plana vitrectomy in Chorioretinitis Sclopetaria has shown considerable effectiveness in improving visual function, retinal reattachment, and macular hole closure in patients with blast-related ocular trauma. The functional results of vitrectomy are better when the sclopetaria is located in the inferior segment of the fundus and when vitrectomy is performed at earlier stages after trauma, especially in patients with concomitant retinal detachment.

## Data Availability

The datasets used and/or analysed during the current study are available from the corresponding author on reasonable request.

## Abbreviations

BCVA: Best-corrected visual acuity
CS: Chorioretinitis Sclopetaria
ILM: Internal limiting membrane
LP: Light perception
NLP: No light perception
PPV: Pars plana vitrectomy
